# Supplementary material for: Ultra-mild bisulfite outperforms existing methods for 5-methylcytosine detection with low input DNA
Source: Nat Commun. 2025 Nov 13;16:9939. doi: 10.1038/s41467-025-66033-y (PMC12615686; doi:10.1038/s41467-025-66033-y)
Supplement: Supplementary file 1 — Supplementary Information [file 41467_2025_66033_MOESM1_ESM.pdf]

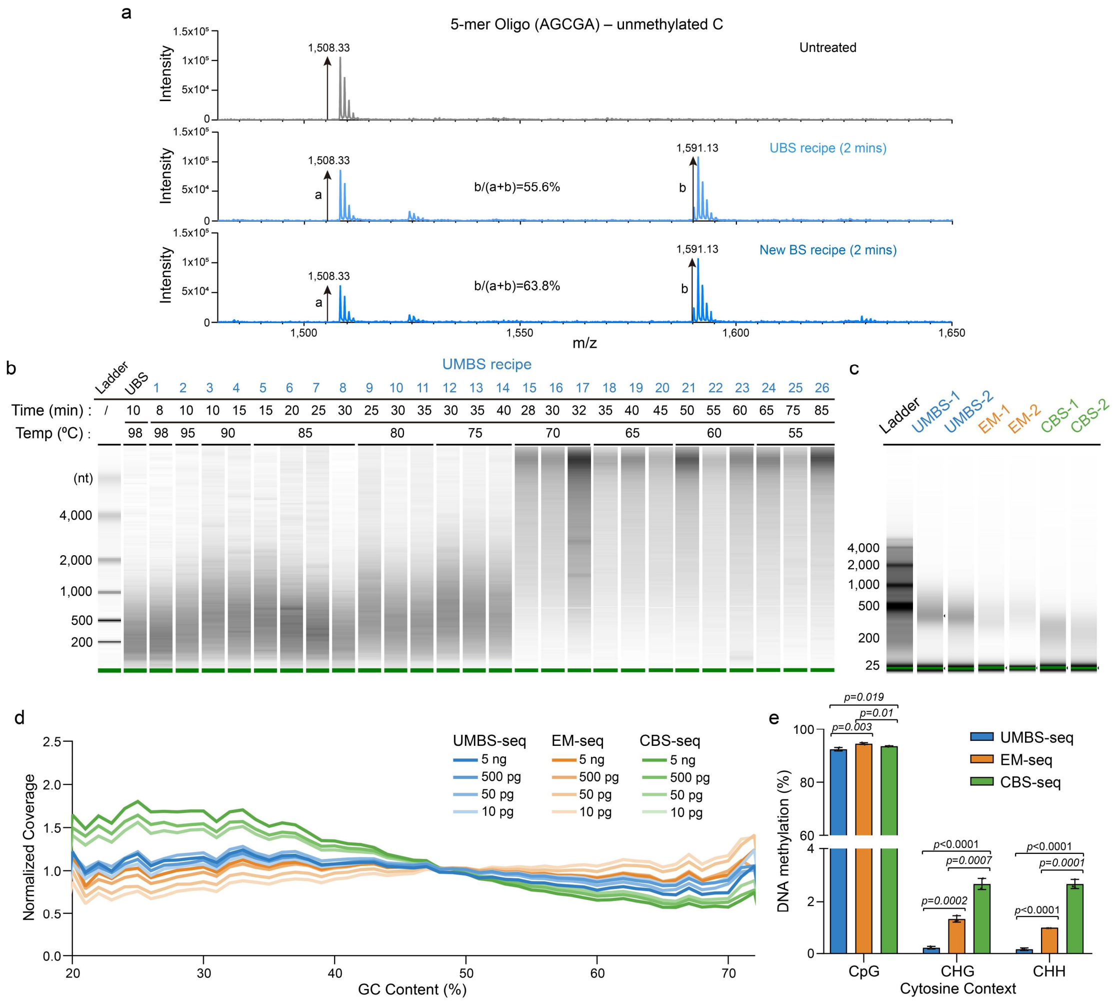

Supplementary Fig. 1: Development of the Ultra-Mild Bisulfite (UMBS) recipe and optimization of UMBS reaction conditions. a, MALDI-TOF MS analysis of unmethylated 5-mer DNA oligonucleotide (AGCGA) treated with Ultra-fast Bisulfite (UBS) and the newly developed bisulfite recipe for 2 minutes. The relative abundances of the unreacted oligo and the U-BS product indicate that the new bisulfite recipe achieves a higher C-to-U conversion efficiency compared to the UBS recipe. **b,** Comparative analysis of DNA damage under varying UMBS reaction conditions, assessed by bioanalyzer (Agilent RNA 6000 Pico). Intact lambda DNA was treated with the UMBS conversion method across a range of temperatures (55 °C to 98 °C) and reaction times (8 to 85 minutes). **c,** TapeStation electropherograms of fragmented, adapter-ligated lambda DNA processed using the three indicated methyl conversion methods. **d,** Normalized GC coverage distribution across the lambda phage genome. Libraries prepared using UMBS-seq and EM-seq (NEB) show significantly more uniform GC coverage compared to those prepared with CBS-seq. **e,** Estimated average DNA methylation levels at CpG sites in methylated pUC19 DNA. UMBS-seq was compared with EM-seq and CBS-seq as benchmarks. Statistical significance was evaluated using two-tailed Student's t test; p-values are indicated. N = 3 libraries per method prepared from the 5ng DNA input were analyzed. Data are presented as mean values +/- SD.

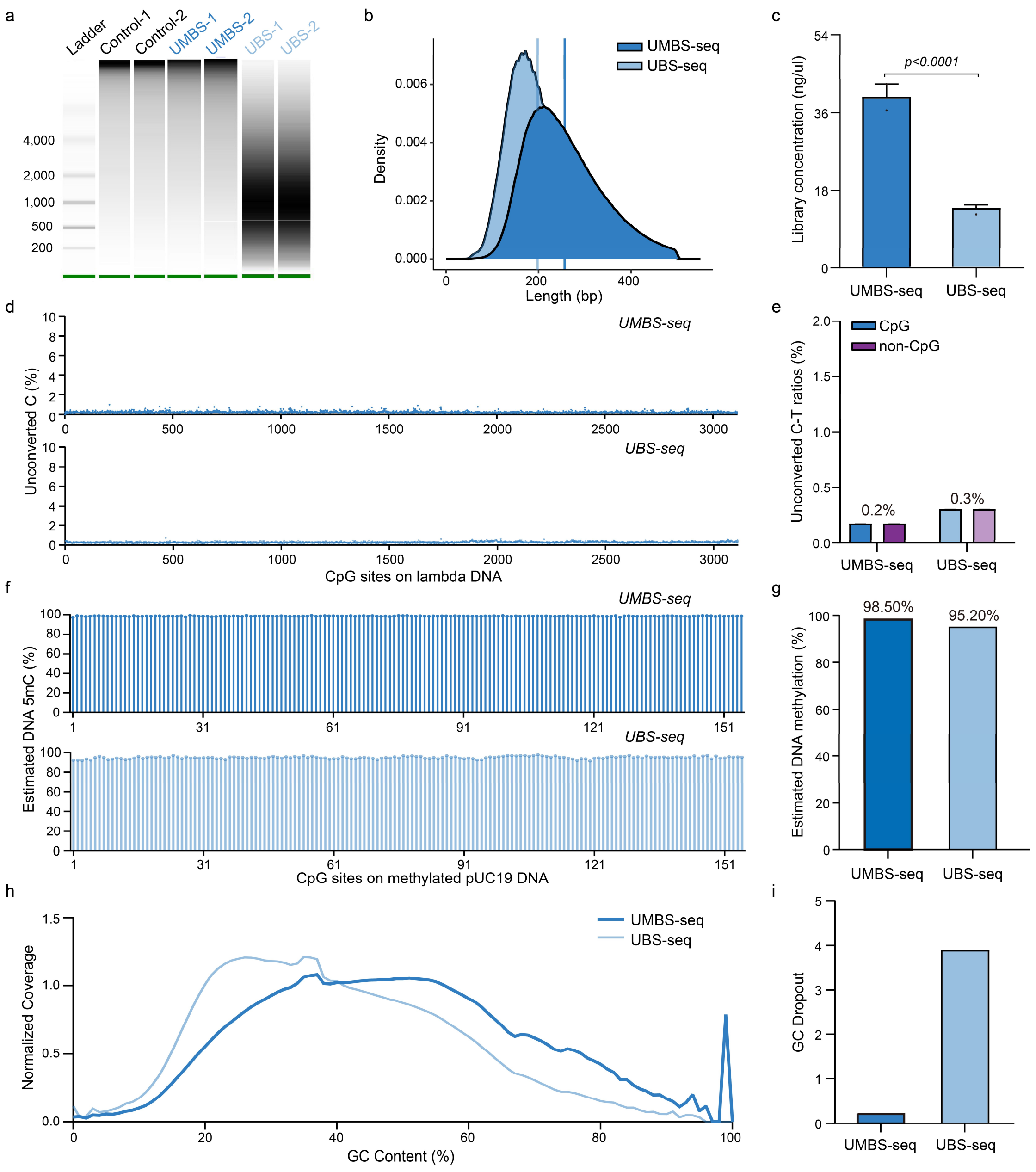

Supplementary Fig. 2: Systematic performance comparison between Ultra-Mild Bisulfite (UMBS) and Ultra-fast Bisulfite (UBS) methyl conversion methods. a, Comparative analysis of DNA damage in 100 ng of intact lambda DNA induced by untreated, UMBS, UBS methyl conversion methods, assessed using a bioanalyzer (Agilent RNA 6000 Pico). **b,** DNA insert size distribution of sequencing libraries prepared from 10 ng mESC gDNA, demonstrating that UMBS-seq preserves longer DNA insert size with reduced DNA damage than UBS-seq. **c,** Concentrations of sequencing libraries generated from the specified lambda DNA amounts after treatment with UMBS and UBS conversion methods. Library concentration was measured after 9 PCR cycles with a 20 µL final elution volume. Statistical significance was assessed using two-tailed Student's t test; p-values are indicated. N = 2 libraries per method prepared from the 10 ng lambda DNA input were analyzed. Data are presented as mean values +/- SD. **d,** Scatterplot showing the unconverted C ratios at individual CpG site of lambda DNA treated with UMBS and UBS conversion methods. **e,** Bar plot shows the averaged unconverted C-T ratios of cytosines in both CpG and non-CpG contexts in lambda DNA, treated with the UMBS-seq and UBS-seq methods. **f,** Cleveland dot plot showing the estimated DNA methylation levels at each CpG site of methylated pUC19 DNA treated with the UMBS-seq

and UBS-seq methods. **g,** Bar plot showing the average DNA methylation levels across all CpG sites in methylated pUC19 DNA treated with the UMBS-seq and UBS-seq methods. **h,** GC-bias plot for UMBS-seq and UBS-seq libraries prepared from 10 ng of mESC gDNA. UMBS-seq shows better GC coverage uniformity compared to UBS-seq. **i,** Bar plot showing the GC dropout ratios in UMBS-seq and UBS-seq libraries prepared using mESC gDNA.

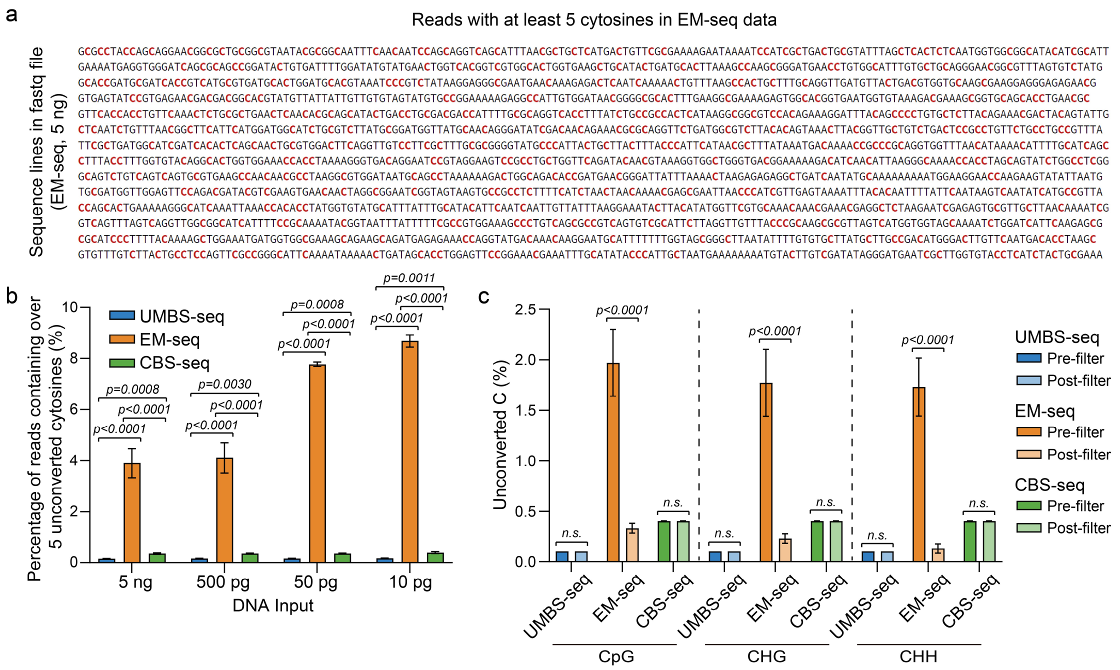

Supplementary Fig. 3: Presence of unconverted reads in Enzymatic Methylation conversion (EM-seq) libraries. a, Screenshot of filtered unconverted reads (containing more than 5 unconverted cytosines) from an EM-seq-treated unmethylated lambda DNA library. Unconverted cytosines are highlighted in red. **b**, Bar plot showing the percentage of reads containing more than 5 unconverted cytosines in lambda DNA libraries prepared with each input and method. **c**, Ratios of unconverted cytosines in lambda DNA libraries pre- and post-filtering of reads containing more than 5 unconverted cytosines. Statistical significance was evaluated using two-tailed Student's t test; p-values are indicated. N = 3 libraries per method, prepared from the same DNA input, were analyzed. Data are presented as mean values +/- SD.

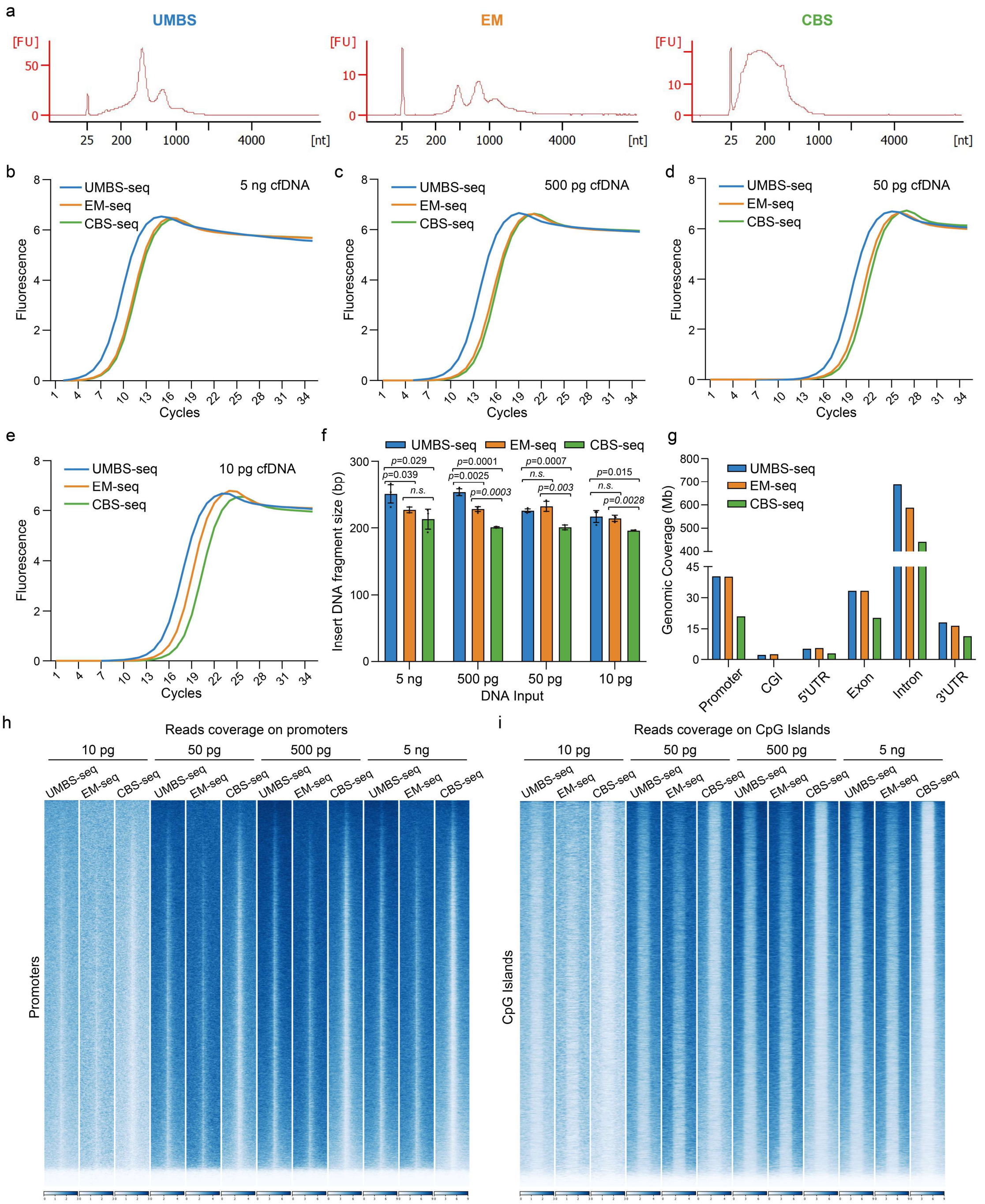

Supplementary Fig. 4: UMBS-seq demonstrates superior performance on low-input cfDNA. a, Electropherogram plots of cfDNA following treatment with the UMBS, EM, and CBS methyl conversion methods. **b-e**, Amplification curves from real-time PCR quantification of cfDNA libraries prepared with 5 ng (**b**), 0.5 ng (**c**), 0.05 ng (**d**), and 0.01 ng (**e**) of cfDNA input using UMBS-seq, EM-seq, and CBS-seq methods. **f**, Bar plot showing the averaged DNA insert size of cfDNA libraries prepared using each conversion method at each input. **g**, Genomic coverage (≥1X) of cfDNA libraries across various genomic features. N=3 Libraries were prepared using 5 ng of cfDNA with each method. Data are presented as mean values +/- SD. **h-i**, Heatmaps showing read coverage distribution within a 2 kb window around transcription start sites (TSS) (**h**) and a 1 kb window around CpG islands (**i**). UMBS-seq and EM-seq libraries exhibit greater and more uniform coverage compared to CBS-seq libraries in both regions.

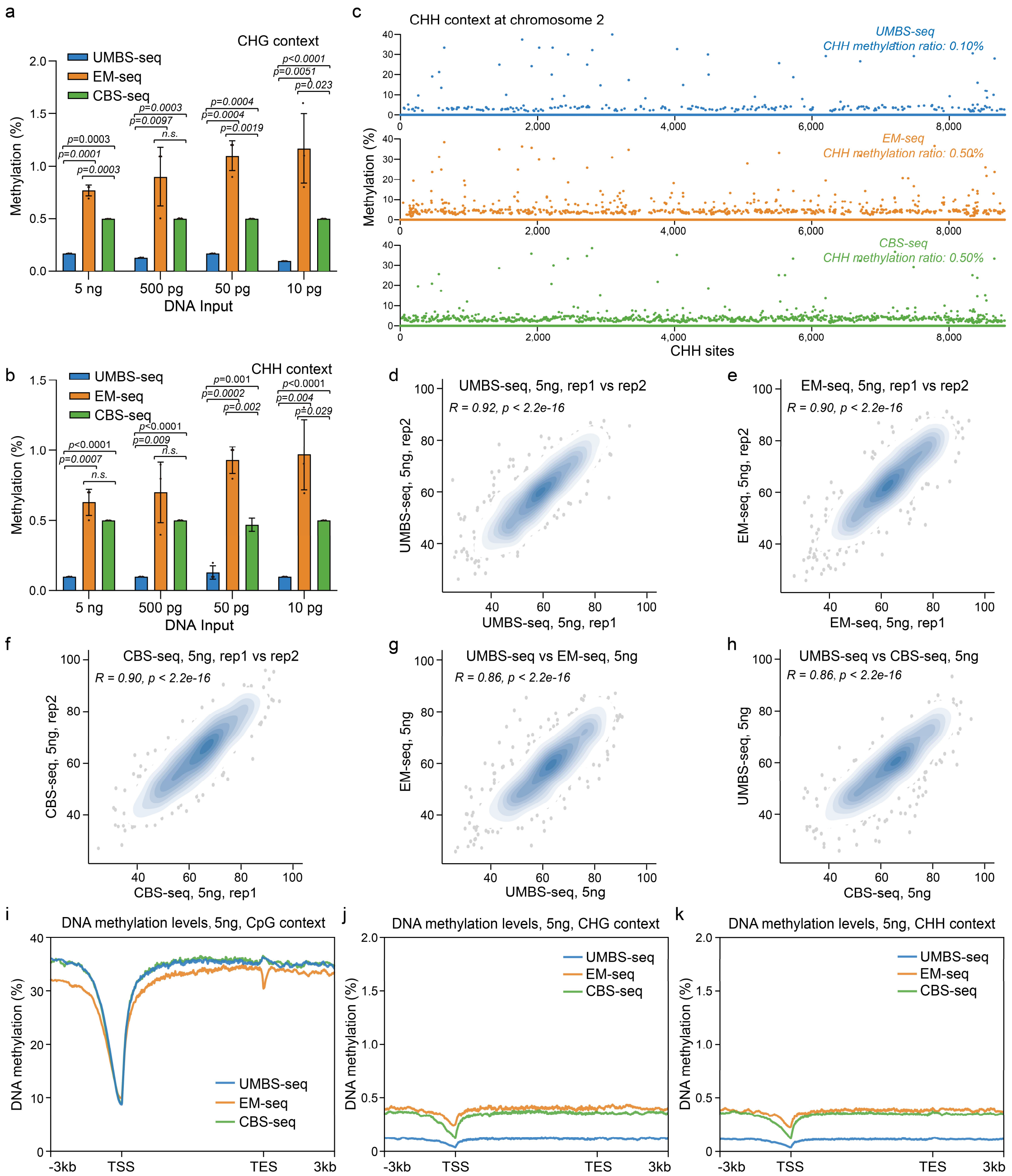

Supplementary Fig. 5: UMBS-seq exhibits lower background in DNA methylation detection from low-input cfDNA. a-b, Estimated average DNA methylation levels at CHG (**a**) and CHH (**b**) contexts in cfDNA libraries prepared with varying cfDNA input amounts. UMBS-seq was compared to EM-seq and CBS-seq as benchmarks. Statistical significance was assessed using two-tailed Student's t test; p-values are indicated. N = 3 libraries per method, prepared from the same DNA input, were analyzed. Data are presented as mean values +/- SD. **c**, Ratios of unconverted cytosines at individual CHH sites (over 10X coverage) on chromosome 2 in cfDNA libraries prepared from 5 ng of input using each method. Average methylation levels across these CHH sites are indicated. **d-f**, Scatter plots showing the correlation of DNA methylation levels at individual CpG sites between technical replicates of UMBS-seq (**d**), EM-seq (**e**), and CBS-seq (**f**) libraries, each prepared from 5 ng of cfDNA input. Correlation coefficients and p-values are indicated. **g-h**, Scatter plots showing the correlation of DNA methylation levels at individual CpG sites in 5 ng cfDNA libraries between UMBS-seq and EM-seq (**g**), and between UMBS-seq and CBS-seq (**h**) libraries. Correlation coefficients and p-

values are indicated. **i**-**k**, Metagene profiles comparing average DNA methylation levels across gene bodies in cfDNA libraries prepared from 5 ng of input using each method. Methylation levels were calculated separately for CpG (**i**), CHG (**j**), and CHH (**k**) contexts.

a

Performance metrics	UMBS-seq	UBS-seq	EM-seq	CBS-seq
Input DNA Range	>=0.01 ng	>=1 ng	>=0.1 ng	>=50 ng
DNA Damage	Low	Medium	Low	Hight
False Positives	Near Zero	Near Zero	High	Moderate
Robustness	Yes	Yes	No	Yes
Protocol Time	2-3 hs	35 mins	7-8 hs	3-4 hs
Library Complexity	High	Medium	High	Low

Supplementary Fig. 6: Comparison of UMBS-seq with UBS-seq, EM-seq, and CBS-seq based on various performance metrics. a, Summary table detailing the comparison of different conversion methods based on input DNA range, DNA damage, false positives, robustness, protocol time, and library complexity.
