# Supplementary material for: Ultra-mild bisulfite outperforms existing methods for 5-methylcytosine detection with low input DNA
Source: Nat Commun. 2025 Nov 13;16:9939. doi: 10.1038/s41467-025-66033-y (PMC12615686; doi:10.1038/s41467-025-66033-y)
Supplement: Supplementary file 5 — Source Data [file 41467_2025_66033_MOESM5_ESM.zip › Source data/Sup Figure 1c, Fragmented Lambda DNA DNA tapstation assay.pdf]

Filename: 2025-07-05 - 16.42.32.HSRNA

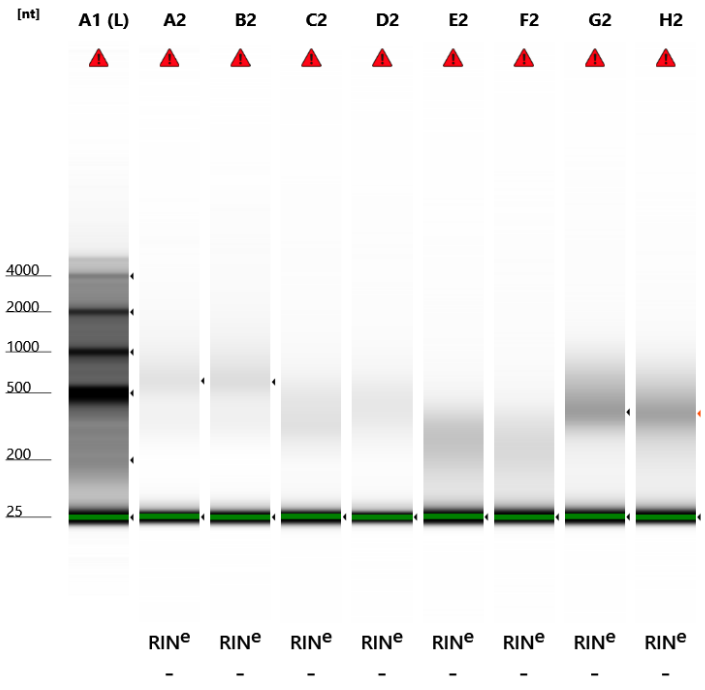

Default image (Contrast 100%)

Sample Info

Well	RINe	28S/18S (Area)	Conc. [pg/µl]	Sample Description	Alert	Observations
A1	-	-	1960	Ladder	⚠	Issue with ladder peak detection (too few peaks detected); Ladder
A2	-	-	109	EK1	⚠	Sample concentration outside functional range for RINe and the assay
B2	-	-	134	EK2	⚠	Sample concentration outside functional range for RINe and the assay
C2	-	-	122	EB1	⚠	Sample concentration outside functional range for RINe and the assay
D2	-	-	113	EB2	⚠	Sample concentration outside functional range for RINe and the assay
E2	-	-	200	UB1	⚠	Sample concentration outside functional range for RINe and the assay
F2	-	-	145	UB2	⚠	Sample concentration outside functional range for RINe and the assay
G2	-	-	304	UM1	⚠	Sample concentration outside functional range for RINe and the assay
H2	-	-	304	UM2	⚠	Sample concentration outside functional range for RINe and the assay

 © Agilent Technologies, Inc. 2023

A1: Ladder

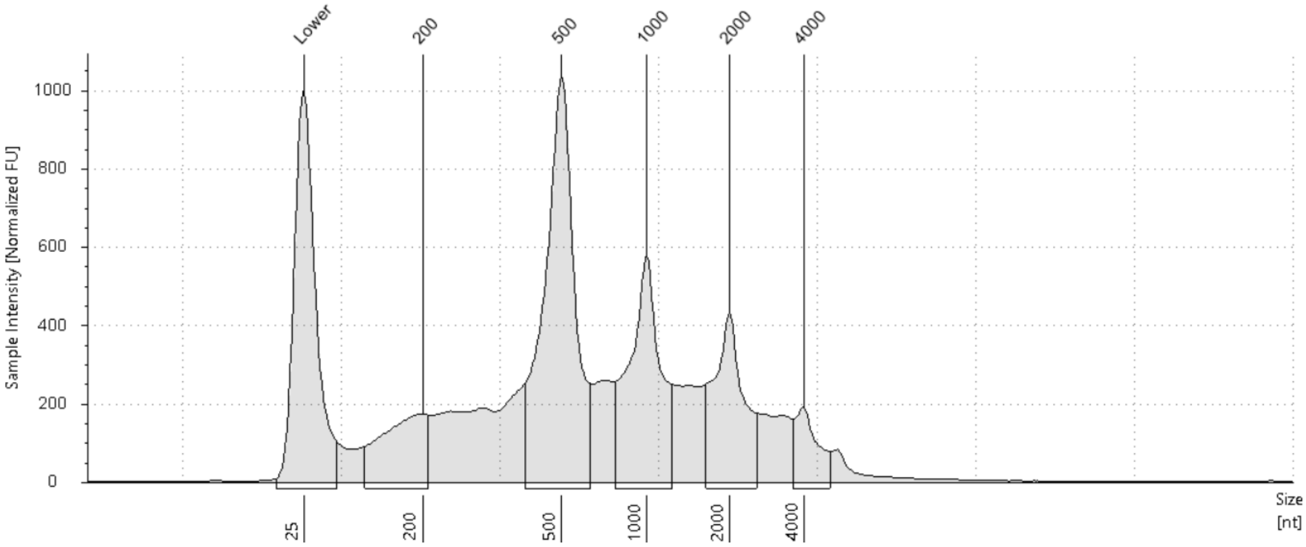

Sample Table

Well	RINe	28S/18S (Area)	Conc. [pg/µl]	Sample Description	Alert	Observations
A1	-	-	1960	Ladder	⚠	Issue with ladder peak detection (too few peaks detected); Ladder

Peak Table

Size [nt]	Calibrated Conc. [pg/µl]	Assigned Conc. [pg/µl]	Peak Molarity [pmol/l]	% Integrated Area	Peak Comment	Observations
25	700	700	82400	-		Lower Marker
200	138	-	2030	10.71		
500	545	-	3200	42.33		
1000	309	-	907	23.97		
2000	220	-	323	17.09		
4000	75.9	-	55.8	5.90		

 © Agilent Technologies, Inc. 2023

A2: EK1

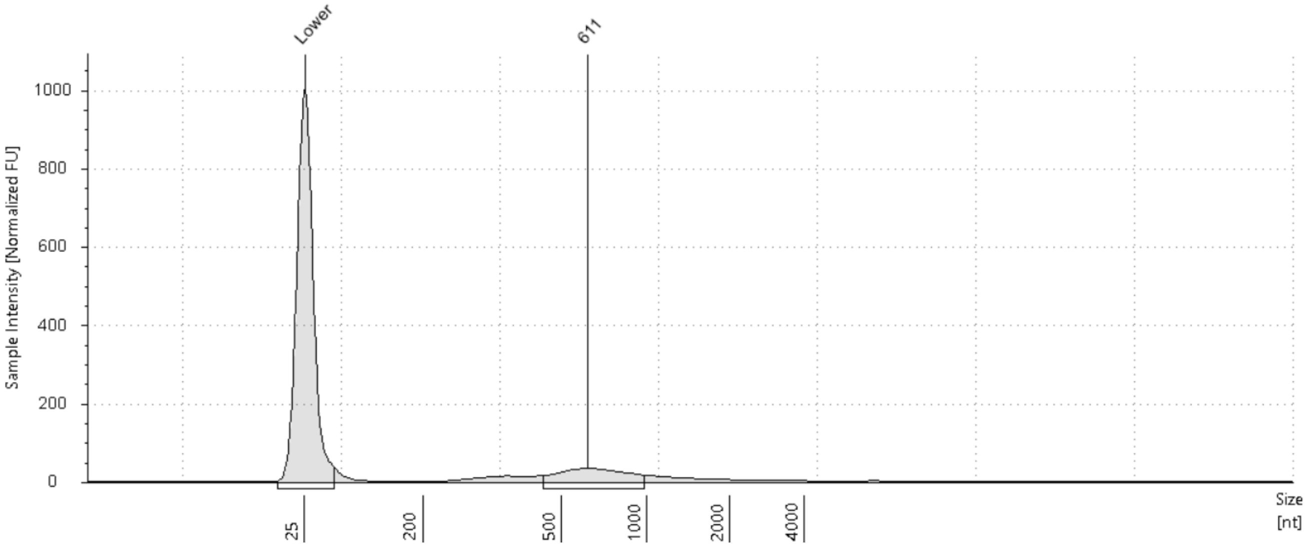

Sample Table

Well	RINe	28S/18S (Area)	Conc. [pg/µl]	Sample Description	Alert	Observations
A2	-	-	109	EK1	⚠	Sample concentration outside functional range for RINe and the assay

Peak Table

Size [nt]	Calibrated Conc. [pg/µl]	Assigned Conc. [pg/µl]	Peak Molarity [pmol/l]	% Integrated Area	Peak Comment	Observations
25	700	700	82400	-		Lower Marker
611	52.7	-	254	100.00		

B2: EK2

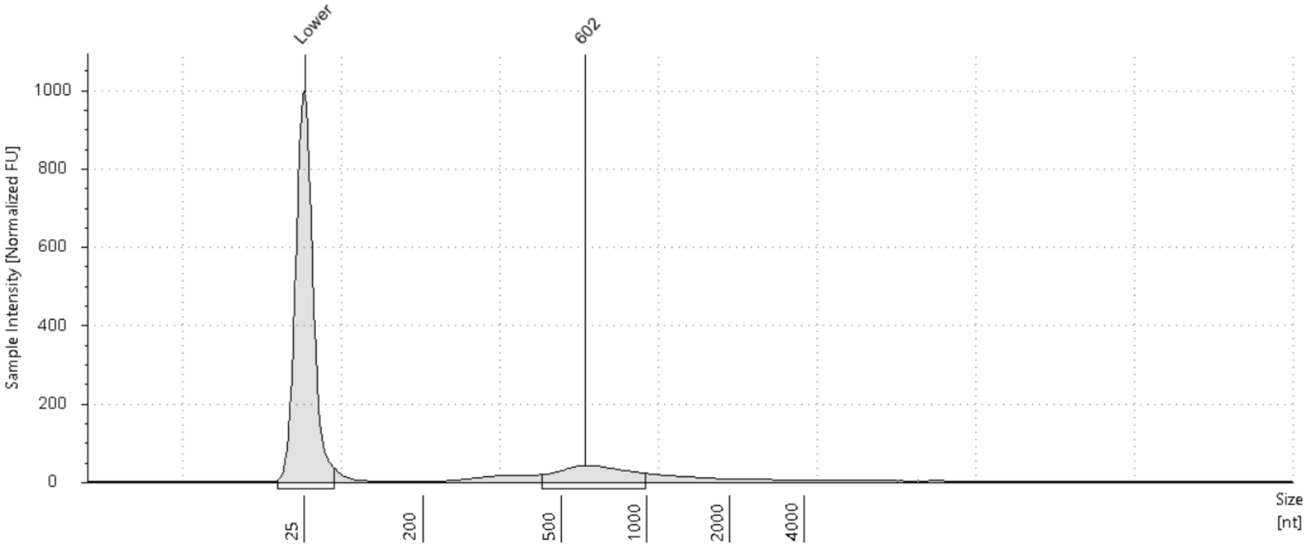

Sample Table

Well	RINe	28S/18S (Area)	Conc. [pg/µl]	Sample Description	Alert	Observations
B2	-	-	134	EK2	⚠	Sample concentration outside functional range for RINe and the assay

Peak Table

Size [nt]	Calibrated Conc. [pg/µl]	Assigned Conc. [pg/µl]	Peak Molarity [pmol/l]	% Integrated Area	Peak Comment	Observations
25	700	700	82400	-		Lower Marker
602	62.3	-	305	100.00		

C2: EB1

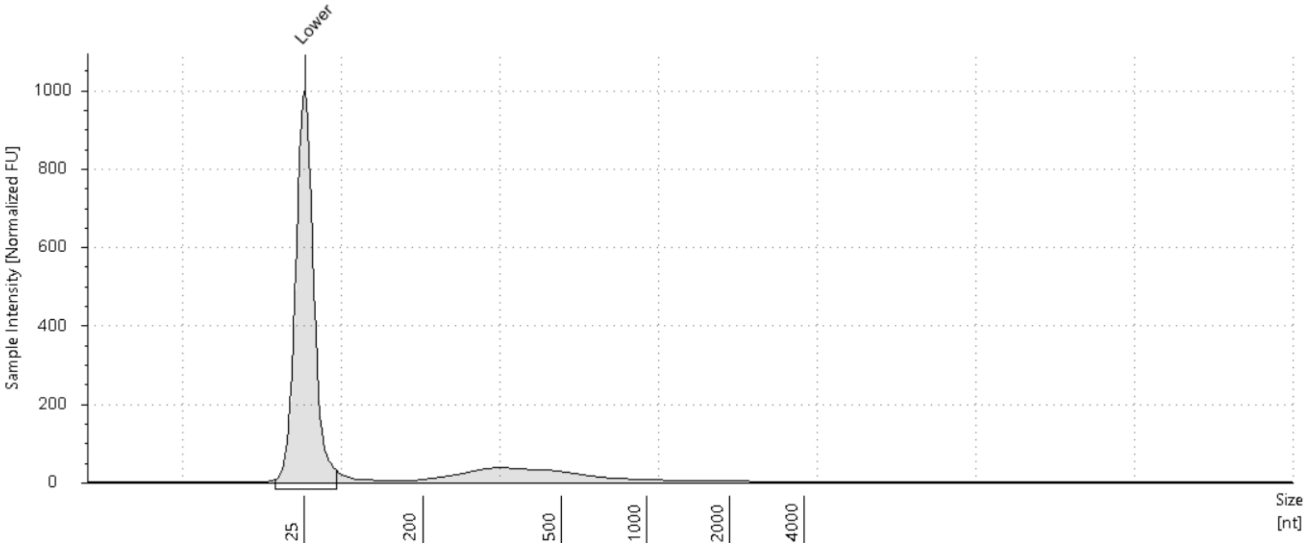

Sample Table

Well	RINe	28S/18S (Area)	Conc. [pg/µl]	Sample Description	Alert	Observations
C2	-	-	122	EB1	⚠	Sample concentration outside functional range for RINe and the assay

Peak Table

Size [nt]	Calibrated Conc. [pg/µl]	Assigned Conc. [pg/µl]	Peak Molarity [pmol/l]	% Integrated Area	Peak Comment	Observations
25	700	700	82400	-		Lower Marker

 © Agilent Technologies, Inc. 2023

D2: EB2

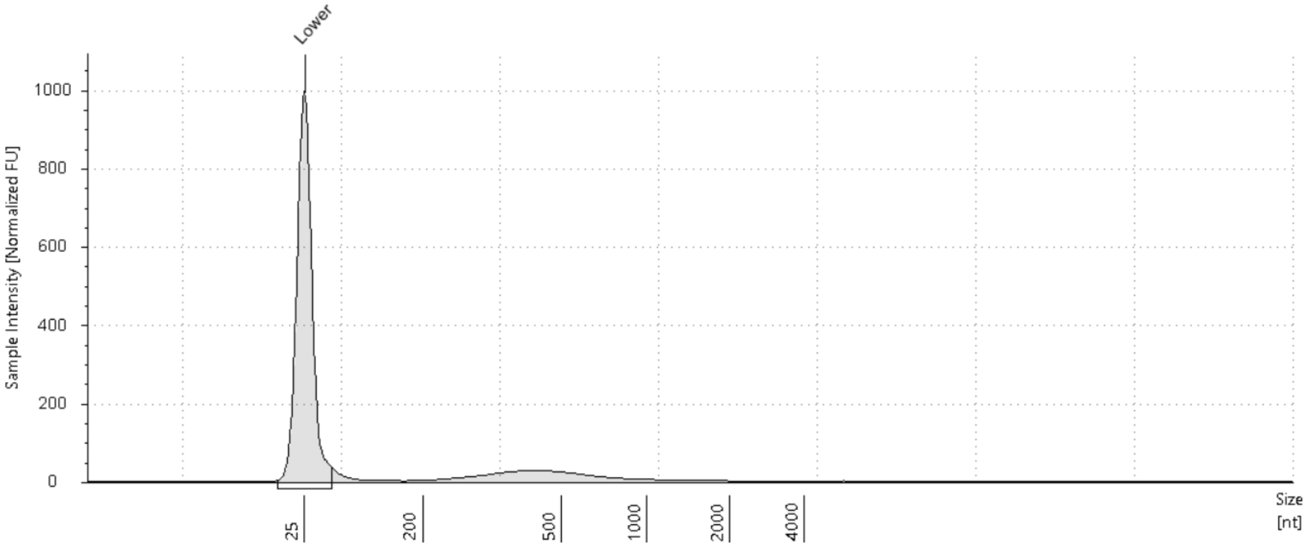

Sample Table

Well	RINe	28S/18S (Area)	Conc. [pg/µl]	Sample Description	Alert	Observations
D2	-	-	113	EB2	⚠	Sample concentration outside functional range for RINe and the assay

Peak Table

Size [nt]	Calibrated Conc. [pg/µl]	Assigned Conc. [pg/µl]	Peak Molarity [pmol/l]	% Integrated Area	Peak Comment	Observations
25	700	700	82400	-		Lower Marker

E2: UB1

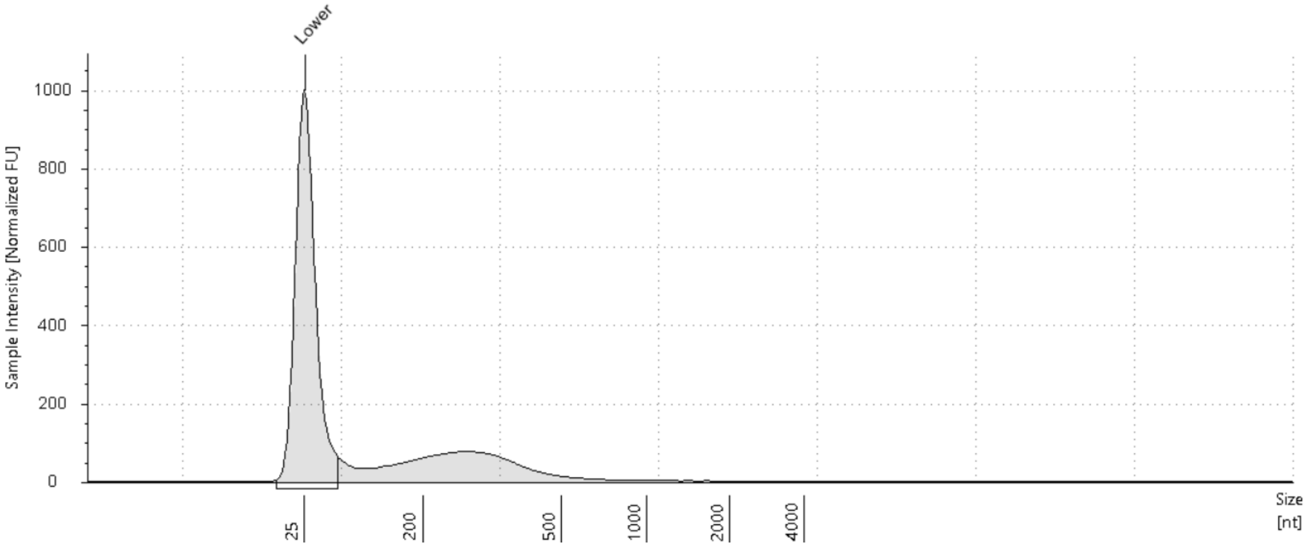

Sample Table

Well	RINe	28S/18S (Area)	Conc. [pg/µl]	Sample Description	Alert	Observations
E2	-	-	200	UB1	⚠	Sample concentration outside functional range for RINe and the assay

Peak Table

Size [nt]	Calibrated Conc. [pg/µl]	Assigned Conc. [pg/µl]	Peak Molarity [pmol/l]	% Integrated Area	Peak Comment	Observations
25	700	700	82400	-		Lower Marker

 © Agilent Technologies, Inc. 2023

F2: UB2

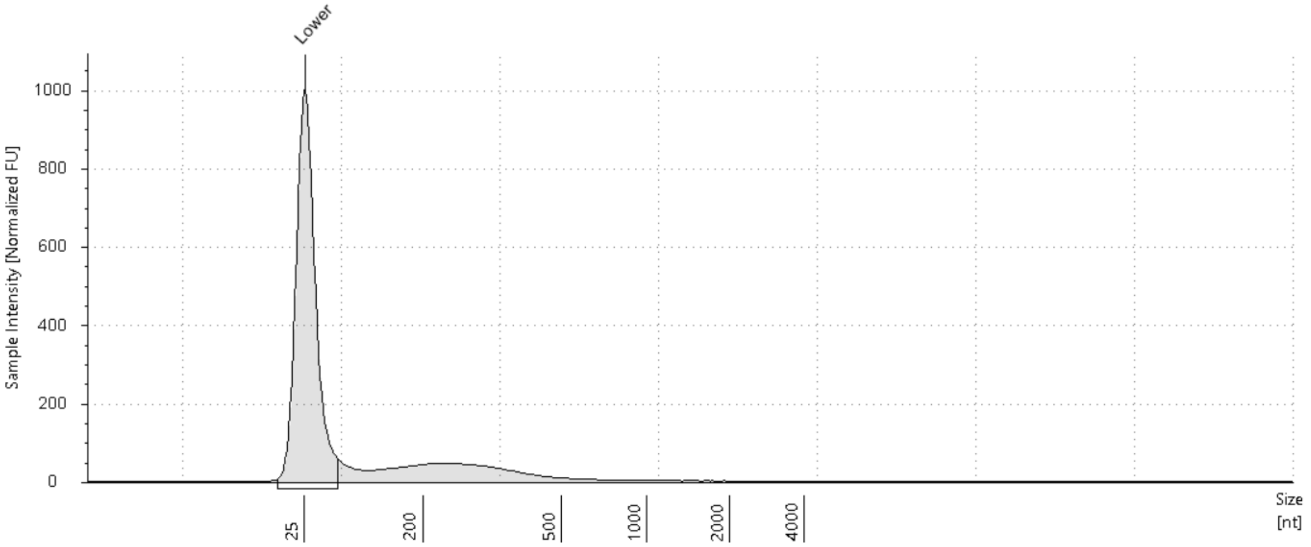

Sample Table

Well	RINe	28S/18S (Area)	Conc. [pg/µl]	Sample Description	Alert	Observations
F2	-	-	145	UB2	⚠	Sample concentration outside functional range for RINe and the assay

Peak Table

Size [nt]	Calibrated Conc. [pg/µl]	Assigned Conc. [pg/µl]	Peak Molarity [pmol/l]	% Integrated Area	Peak Comment	Observations
25	700	700	82400	-		Lower Marker

 © Agilent Technologies, Inc. 2023

G2: UM1

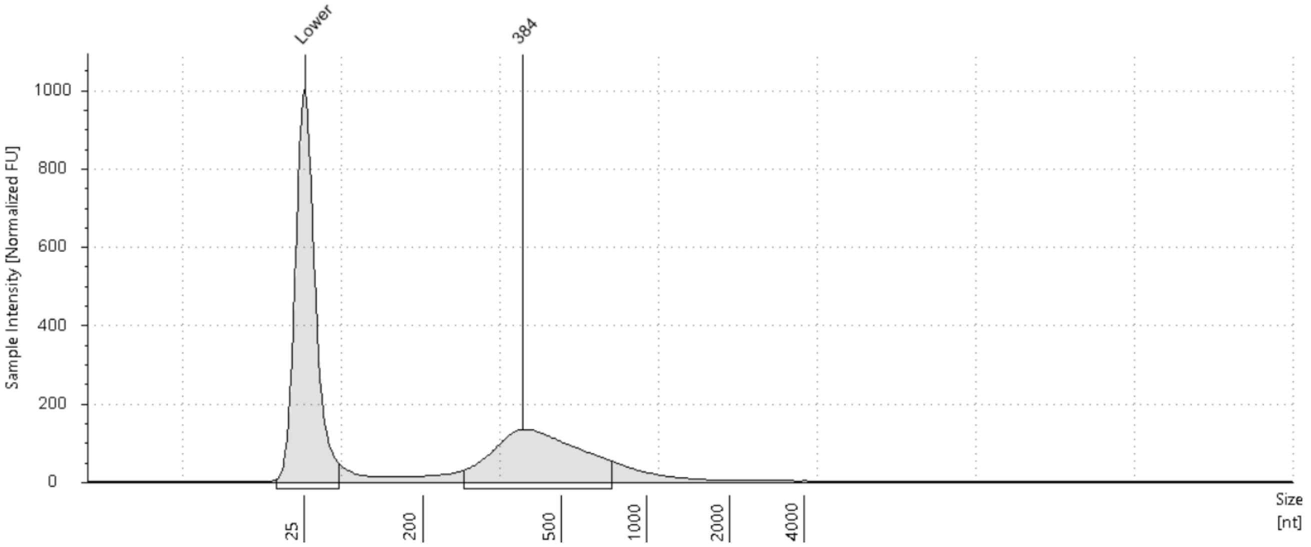

Sample Table

Well	RINe	28S/18S (Area)	Conc. [pg/µl]	Sample Description	Alert	Observations
G2	-	-	304	UM1	⚠	Sample concentration outside functional range for RINe and the assay

Peak Table

Size [nt]	Calibrated Conc. [pg/µl]	Assigned Conc. [pg/µl]	Peak Molarity [pmol/l]	% Integrated Area	Peak Comment	Observations
25	700	700	82400	-		Lower Marker
384	221	-	1690	100.00		

 © Agilent Technologies, Inc. 2023

H2: UM2

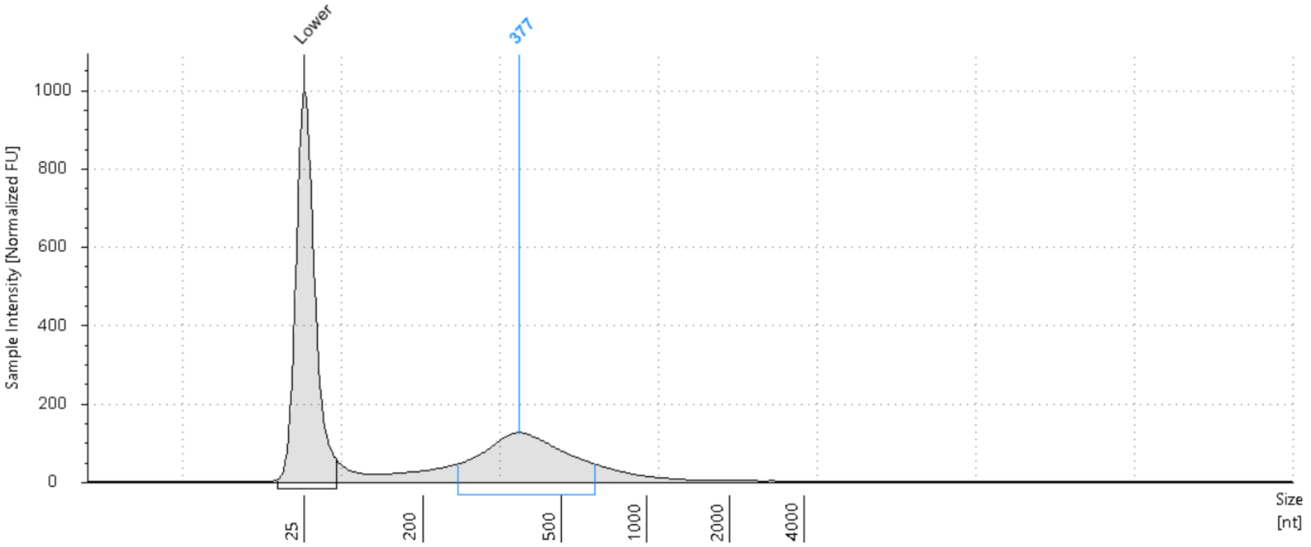

Sample Table

Well	RINe	28S/18S (Area)	Conc. [pg/µl]	Sample Description	Alert	Observations
H2	-	-	304	UM2	⚠	Sample concentration outside functional range for RINe and the assay

Peak Table

Size [nt]	Calibrated Conc. [pg/µl]	Assigned Conc. [pg/µl]	Peak Molarity [pmol/l]	% Integrated Area	Peak Comment	Observations
25	700	700	82400	-		Lower Marker
377	204	-	1590	100.00		

 © Agilent Technologies, Inc. 2023
