# Supplementary material for: Ultra-mild bisulfite outperforms existing methods for 5-methylcytosine detection with low input DNA
Source: Nat Commun. 2025 Nov 13;16:9939. doi: 10.1038/s41467-025-66033-y (PMC12615686; doi:10.1038/s41467-025-66033-y)
Supplement: Supplementary file 5 — Source Data [file 41467_2025_66033_MOESM5_ESM.zip › Source data/Figure 1b, MALDI figures, UMBS can't convert 5mC oligos.zip!/MALDI figures, UMBS can't convert 5mC oligos/C-UMBS-Spectrum, MALDI.pdf]

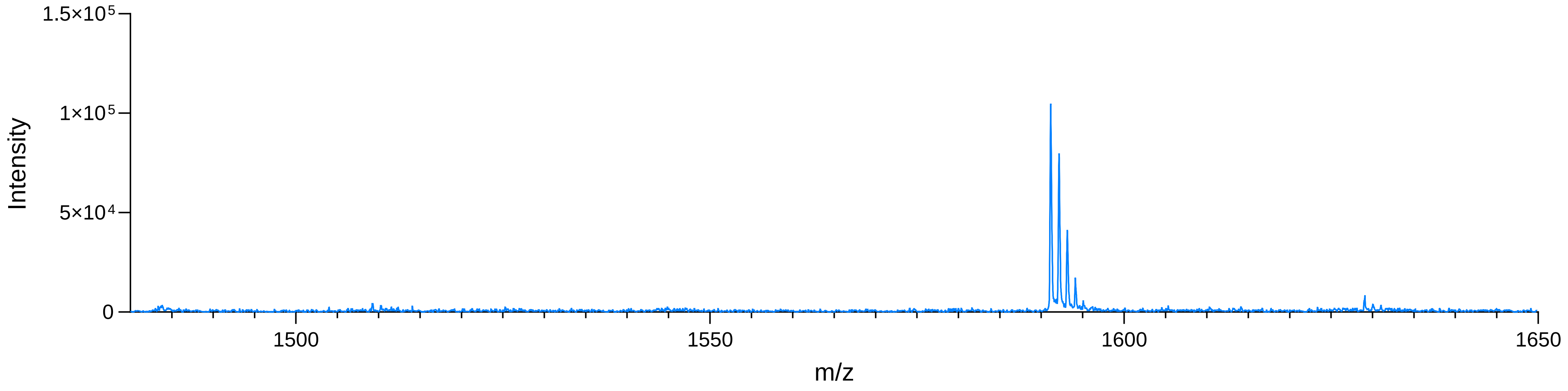
1.5×10^5
1×10^5
5×10^4
0
Intensity
1500
1550
1600
1650
m/z
