# Supplementary material for: Ultra-mild bisulfite outperforms existing methods for 5-methylcytosine detection with low input DNA
Source: Nat Commun. 2025 Nov 13;16:9939. doi: 10.1038/s41467-025-66033-y (PMC12615686; doi:10.1038/s41467-025-66033-y)
Supplement: Supplementary file 5 — Source Data [file 41467_2025_66033_MOESM5_ESM.zip › Source data/Sup Figure 1a, MALDI figures, UMBS recipe showed higher BS efficiency than UBS-seq.zip!/MALDI figures, UMBS recipe showed higher BS efficiency than UBS-seq/Control_2min_spectrum, MALDI.pdf]

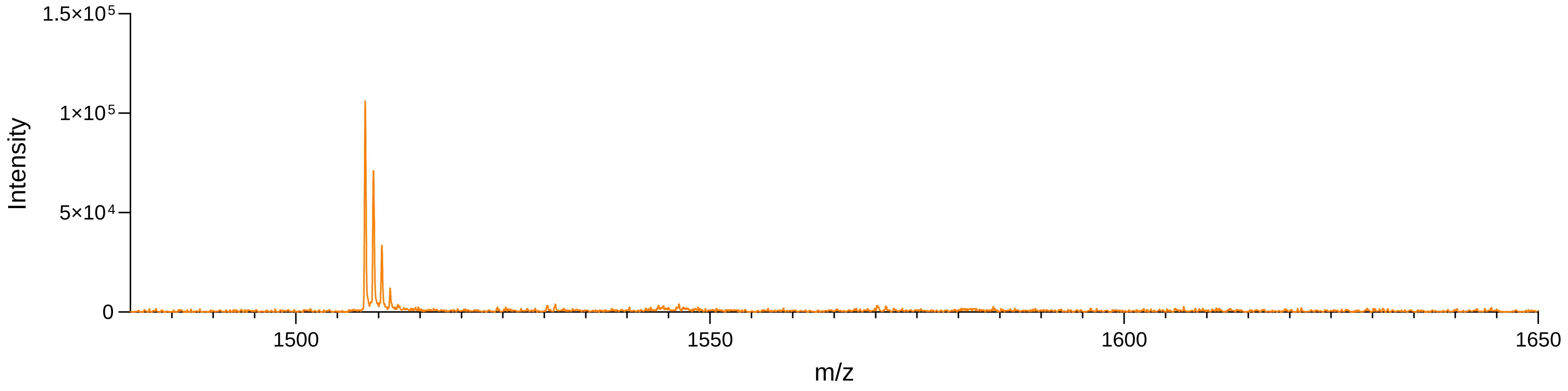

1.5×10⁵
1×10⁵
5×10⁴
0
Intensity
1500
1550
1600
1650
m/z
